# Multiple Phagemids Within Single Bacterial Colonies During Construction and Bio-Panning of Single-Domain (VH and VHH) Phage Display Libraries: A Retransformation Strategy for Reliable Clone Screening

**DOI:** 10.3390/biom16101523

**Published:** 2026-10-19

**Authors:** Kyeongeun Kim, Wookyoung Chun, Hyunji Yang, Yonghee Lee, Junho Chung

**Affiliations:** 1 Department of Biochemistry and Molecular Biology, Seoul National University College of Medicine, Seoul 03080, Republic of Korea; 2 Cancer Research Institute, Seoul National University College of Medicine, Seoul 03080, Republic of Korea; 3 Interdisciplinary Program in Cancer Biology Major, Seoul National University College of Medicine, Seoul 03080, Republic of Korea; 4 Department of Biomedical Science, Seoul National University College of Medicine, Seoul 03080, Republic of Korea; 5 BioMonster Inc., Samsung Cancer Research Building, Seoul National University, Seoul 03080, Republic of Korea

**Keywords:** single-domain antibody (sdAb), variable domain of the heavy chain of a heavy-chain-only antibody (VHH), variable domain of the heavy chain (VH), phage display, colony-level clonality

## Abstract

Background/Objectives: Phage display relies on the physical linkage between the antibody fragment displayed on a virion (phenotype) and the phagemid encoding it (genotype), allowing antigen-binding clones enriched by bio-panning to be identified by sequencing of individual colonies. In colony-based screening, an additional practical assumption is that each isolated bacterial colony represents a single phagemid sequence. Single-domain antibodies (sdAbs), including the variable domain of the heavy chain of a heavy-chain-only antibody (VHH), are increasingly used as displayed molecules, yet their library construction and bio-panning protocols have been adopted, largely unmodified, from those established for single-chain variable fragment (scFv) and fragment antigen-binding (Fab). We therefore asked whether the underlying assumption of one phagemid per colony holds for sdAb phage display. Methods: Using two alpaca immune libraries—a VHH library and a VH/VHH library (VH, the variable domain of the heavy chain)—we tracked Sanger sequencing chromatograms through library construction and three rounds of bio-panning, and compared classical single-colony screening with a strategy in which pooled phagemid DNA recovered after bio-panning was retransformed into naïve *Escherichia coli* (*E. coli*) before screening. Results: Mixed-peak chromatograms were infrequent in the initial libraries (10–20%) but accounted for 80% and 50% of chromatograms in the final bio-panning round of the VHH and VH/VHH libraries. Classical screening—periplasmic enzyme-linked immunosorbent assay (ELISA) followed by Sanger sequencing—failed: only 5.7% and 6.9% of antigen-binding colonies yielded interpretable single-peak sequences. Retransformation restored clonality, raising the proportion of single-peak chromatograms to ~88% overall, and retransformation of individual mixed colonies showed that single colonies harbored multiple distinct phagemids. Conclusions: Colony-level clonality is not strictly preserved in single-domain VH and VHH phage display—more plausibly through multiple phage infection during amplification—and a simple retransformation step restores reliable identification of antigen-binding clones.

## 1. Introduction

Phage display technology was first introduced by Smith in 1985 as a method for displaying polypeptides or proteins on the surface of filamentous bacteriophage [1]. Building on this work, McCafferty et al. demonstrated that immunoglobulin variable domains could be functionally displayed on filamentous phage while retaining antigen-binding specificity, and that antigen-binding clones could be efficiently enriched through iterative rounds of affinity selection [2]. This contribution was recognized by the 2018 Nobel Prize in Chemistry, which was awarded jointly to George P. Smith and Sir Gregory P. Winter for the phage display of peptides and antibodies.

To date, more than 70 phage display library-derived antibodies have entered clinical studies, of which 16 have received U.S. Food and Drug Administration (FDA)/European Medicines Agency (EMA) approval—including adalimumab, ranibizumab, and necitumumab—with dozens more currently in clinical development [3,4,5]. Adalimumab (Humira^®^, AbbVie), a tumor necrosis factor-α (TNF-α) inhibitor approved by the FDA in 2002, was the first phage display library-derived antibody to receive approval; the most recent addition is cosibelimab (Unloxcyt^®^), a programmed death-ligand 1 (PD-L1)-targeting antibody approved by the FDA in 2024. These approved antibodies now span a broad therapeutic landscape, extending beyond oncology to inflammatory, autoimmune, ophthalmic, and infectious disease indications [3,5]. Phage display therefore remains not only a foundational molecular biology technique but also an innovative platform that continues to drive therapeutic antibody discovery.

More recently, phage display library formats have diversified beyond the single-chain variable fragment (scFv) and fragment antigen-binding (Fab) formats to include single-domain antibody (sdAb) [6]. Both scFv and Fab comprise the variable domains of the heavy (V_H_) and light (V_L_) chains, joined either by a peptide linker or by a disulfide bond between the constant domain 1 of the heavy chain (C_H_1) and constant domain of the light chain (C_L_). Although scFv and Fab fragments are widely used as antibody discovery formats and are amenable to bacterial expression, their screening behavior may not fully reflect the properties of immunoglobulin G (IgG), the most common form of therapeutic antibodies [7]. In particular, scFv candidates can exhibit format-dependent differences in folding, aggregation, and soluble expression, which may influence their apparent performance during screening [8,9]. Importantly, improvements in antigen binding identified in scFv format are not always retained after conversion to IgG [10]. Fragment-derived candidates should therefore be re-evaluated for both antigen binding and developability in their final format, such as IgG [11,12].

In 1993, Hamers-Casterman and colleagues identified a class of heavy-chain-only immunoglobulins in the serum of the dromedary camel (*Camelus dromedarius*) [13]. These heavy-chain-only antibodies rely solely on the variable domain of the heavy chain of a heavy-chain-only antibody (VHH) for antigen binding, with the absence of C_H_1 compensated for by an extended hinge region in certain IgG subclasses. VHH offers several biophysical advantages over the variable fragment (Fv) unit as an antigen-binding module [14]. First, VHH is roughly half the size of Fv, with a molecular weight of approximately 12–15 kDa [15]; this compact size reflects a key structural difference in framework region (FR) 2, where the hydrophobic residues that mediate the V_H_–V_L_ interface are replaced in VHH by smaller, polar residues, eliminating the hydrophobic patch responsible for Fv self-association and aggregation. As a result, VHH exhibits greater thermodynamic stability, resistance to chemical denaturants and proteolytic degradation, and tolerance to extremes of pH and ionic strength [15,16]. Second, its smaller size enables more efficient penetration into solid tumors and across the blood–brain barrier [17]. Third, its comparatively long complementarity-determining region 3 (CDR3) loop confers a convex paratope capable of accessing grooves and clefts on antigen surfaces—such as G protein-coupled receptor (GPCR) [18] and viral glycoproteins [19]—that are largely inaccessible to Fv. Fourth, because VHH functions as a single, self-sufficient polypeptide, its production is considerably simpler and less costly [20]. A further important advantage of VHHs becomes apparent during the construction of immune phage display libraries. In each conventional B cell, the antigen-binding site is encoded by a cognate V_H_–V_L_ pair. In standard combinatorial scFv or Fab library construction, however, B cells are pooled and lysed, after which V_H_ and V_L_ transcripts are amplified separately and reassembled by overlap-extension PCR or sequential cloning into a phagemid vector [21]. Because the original cellular linkage between the heavy- and light-chain sequences is lost during this process, V_H_ and V_L_ domains derived from different B cells can be assembled into non-cognate combinations [22]. Random V_H_–V_L_ pairing may eliminate genuine binders present in the original immune repertoire or generate artificial binders that were not produced in vivo, thereby altering the sensitivity and specificity of antibody discovery. VHH libraries avoid this limitation because each VHH sequence encodes a complete antigen-binding domain and can be cloned directly into the phage display vector without the separation and reassociation of heavy- and light-chain repertoires [20]. Thus, VHH-based library construction removes the uncertainty associated with reconstructing native V_H_–V_L_ pairing.

These combined advantages have already been translated into clinical success: caplacizumab, a VHH-based therapeutic, was approved in the European Union in 2018 and the US in 2019 for the treatment of acquired thrombotic thrombocytopenic purpura (aTTP) [23,24].

Caplacizumab’s development via phage display and its approval confirmed that this platform can be effectively applied to the development of VHH antibodies. Despite these distinct structural and biophysical advantages, the construction of VHH phage display libraries and their bio-panning procedures have largely been adapted from protocols originally established for the phage display of scFv and Fab, with minimal modification [25,26,27].

Phage display enables the construction of antibody libraries with extensive diversity, typically comprising 10^6^–10^11^ distinct variants within a population exceeding 10^12^ phage particles [28]. Such immune libraries have been generated from a range of immunized species, including mice [29], chickens [30], rabbits [31], sheep [32], cattle [33], non-human primates [34], and humans [35,36]. Naïve or fully synthetic human libraries can also be constructed without prior immunization, enabling the direct isolation of human or humanized antibodies. In each case, the peptide or protein of interest is genetically fused to a phage coat protein, most commonly pIII or pVIII, creating a physical linkage between the displayed phenotype and its encoding genotype [1,37]. This genotype–phenotype linkage enables repeated affinity selection and amplification, making phage display a powerful platform for antibody discovery and protein engineering [38].

Bio-panning, the procedure used to enrich antigen-specific binders from this diverse repertoire, consists of iterative cycles of phage incubation with an immobilized target, followed by washing, elution, and amplification of bound phage for re-selection [39,40]. The enriched phage population is then used to infect host cells, which are plated to obtain individual colonies [40]. Individual colonies are cultured for small-scale periplasmic expression of antibody fragments, and their antigen-binding activity is evaluated by periplasmic enzyme-linked immunosorbent assay (ELISA) or phage ELISA to identify antigen-binders [40,41]. Antigen-binders are subjected to Sanger sequencing, generally under the assumption that each colony contains a single phagemid sequence [40].

The principle enabling the enrichment of antigen-binders through bio-panning is “genotype–phenotype linkage” [1,2], in which the antibody fragment displayed on an individual phage particle is encoded by the phagemid packaged within that same particle. Sequence-based screening of individual bacterial colonies requires an additional practical assumption: that each colony represents a clonal phagemid population. During VHH phage display library construction, bio-panning, and selection of antigen-binders by phage or periplasmic ELISA, we observed loss of clonality at the bacterial colony level, with individual colonies containing multiple distinguishable phagemids. 

Therefore, in this study, we traced the occurrence of clonal heterogeneity at the colony level across VH/VHH phage display library construction and the bio-panning process and evaluated retransformation as a strategy for recovering sequence-defined clones.

## 2. Materials and Methods

### 2.1. Expression and Purification of Recombinant Protein

The gene encoding an anti-SARS-CoV-2 receptor-binding domain (RBD)-specific human scFv (clone 27-60) was subcloned into the pCEP4 mammalian expression vector (Invitrogen, Carlsbad, CA, USA; Cat. No. V04450) for the expression of a recombinant scFv-human IgG1 Fc fusion protein with a C-terminal hemagglutinin (HA) tag (scFv-hFc-HA) as described previously [36]. The gene encoding an anti-SARS-CoV-2 RBD-specific human scFv (clone 43-09) was subcloned into the pCEP4 vector for the expression of a recombinant scFv-human IgG1 Fc fusion protein without the HA tag (scFv-hFc) as described previously [36]. Expi293F™ cells (Gibco, Grand Island, NY, USA; Cat. No. A14527) were transiently transfected with the vector using polyethylenimine (PEI) MAX^®^ Transfection Grade Linear Polyethylenimine Hydrochloride (MW 40,000) (Polysciences, Warrington, PA, USA; Cat. No. 24765-1) at a PEI:DNA ratio of 2:1 (*w*/*w*). Transfected cells were cultured in FreeStyle™ 293 Expression Medium (Gibco, Grand Island, NY, USA; Cat. No. 12338018) supplemented with Penicillin–Streptomycin (10,000 U/mL) (Gibco, Grand Island, NY, USA; Cat. No. 15140122; final concentration 100 U/mL penicillin, 100 μg/mL streptomycin) at 37 °C, 5% CO_2_, with orbital shaking at 125 rpm for 7 days. Culture supernatant was harvested by centrifugation at 4000× *g* for 20 min at 4 °C and filtered through a 0.2-μm Acrodisc^®^ syringe filter with a Supor^®^ polyethersulfone (PES) membrane (Pall Corporation, Port Washington, NY, USA; Cat. No. 4612). The recombinant proteins were purified from the supernatant by affinity chromatography using a MabSelect™ affinity chromatography column (Cytiva, Uppsala, Sweden; Cat. No. 17519901) following the manufacturer’s instructions. The recombinant proteins were eluted with Pierce™ IgG Elution Buffer, pH 2.8 (Thermo Scientific, Rockford, IL, USA; Cat. No. 21004) and immediately neutralized with 1 M Tris-HCl, pH 9.0, prepared in house from Tris base (Millipore, Merck KGaA, Darmstadt, Germany; Cat. No. 1.08387).

### 2.2. Immunization and Blood Sampling

One alpaca (*Vicugna pacos*) was immunized by subcutaneous injection with the recombinant 27-60 scFv-hFc-HA fusion protein formulated with Freund’s Adjuvant, Incomplete (Sigma-Aldrich, St. Louis, MO, USA; Cat. No. F5506-10ML), Monophosphoryl lipid A from *Salmonella minnesota* R595 (MPLA-SM) (InvivoGen, San Diego, CA, USA; Cat. Code tlrl-mpla), and QS-21 (GlpBio Technology Inc., Montclair, CA, USA; Cat. No. GC63377) [42]. For each immunization, the antigen solution was adjusted to a final volume of 500 μL with phosphate-buffered saline (PBS; 20× stock, Biosesang, Seongnam, Republic of Korea; Cat. No. PR2007-100-00; diluted to 1× with distilled water). Separately, an adjuvant mixture was prepared by combining 50 μL each of 1 mg/mL MPLA-SM and 1 mg/mL QS-21, plus 400 μL of Freund’s Adjuvant, Incomplete. The antigen solution (500 μL) and the adjuvant mixture (500 μL) were then mixed at a 1:1 (*v*/*v*) ratio and emulsified to produce a stable water-in-oil emulsion with a final volume of 1 mL.

A total of eight injections were administered at two-week intervals. Two weeks after the final immunization, 50 mL of peripheral blood (PB) was collected into five 10-mL BD Vacutainer^®^ K2EDTA blood collection tubes (BD, Franklin Lakes, NJ, USA; Cat. No. 367525).

For high-throughput sequencing (HTS) analysis for the selection of P3 forward primers, 37 PB samples (50 mL per sample) were drawn from alpacas. All procedures involving the alpaca were approved by the Institutional Animal Care and Use Committee (IACUC) of Seoul National University (Approval No. SNU-240930-1, approval date: 16 December 2024; Approval No. SNU-240930-1-1, renewal approval date: 22 December 2025) and were performed in accordance with the institutional guidelines.

### 2.3. Preparation of RNA from Peripheral Blood Mononuclear Cells

From 50 mL of PB sample, peripheral blood mononuclear cells (PBMCs) and plasma were separated by density-gradient centrifugation using Ficoll-Paque™ PLUS density gradient medium (Cytiva, Uppsala, Sweden; Cat. No. 17-1440-02) as described previously [36]. Total RNA was extracted from isolated PBMCs using TRIzol™ Reagent (Invitrogen, Carlsbad, CA, USA; Cat. No. 15596026) following the manufacturer’s instructions.

### 2.4. Primer Design for Alpaca VH/VHH Phage Display Library

Primer design was initiated from a previously reported camelid VHH primer set, which was used as the initial template [25]. The forward primer of this template, 5′-CAGKTGCAGCTCGTGGAGTCNGGNGG-3′, anneals to FR1 and contains degenerate positions (K = G or T; N = A, C, G, or T), corresponding to 32 possible unique primer sequences. To determine which of these sequences were predominantly utilized for library construction, alpaca VHH repertoires were amplified using this degenerate forward primer and subjected to HTS analysis, as described in a later section. This analysis demonstrated that 10 of the 32 possible unique sequences were actually used for amplification, and these 10 primers were selected as the P3 forward primer set (Supplementary Table S1).

To additionally cover the conventional VH repertoire, further forward primers were designed on the basis of the alpaca (*Vicugna pacos*) germline immunoglobulin heavy-chain variable (IGHV) gene sequences deposited in the international ImMunoGeneTics information system (IMGT) database (IMGT/GENE-DB; https://www.imgt.org). Of the 73 IGHV germline genes annotated as functional, 21 were not covered by the P3 primer set and were therefore targeted by newly designed forward primers. All forward primers were designed to anneal at the 5′ end of the V gene so that the entire variable domain (FR1–FR4) could be amplified. To balance the annealing temperatures within each set, primer lengths were adjusted so that the difference in annealing temperature among the primers remained below 5 °C. Through this length adjustment, the ten P3 forward primers were consolidated into two primers (P4-F1 and P4-F2), and five additional primers targeting the remaining VH genes were newly designed, resulting in a total of seven unique forward primers (P4 forward primer set; Supplementary Table S1).

The same strategy was applied to the design of the reverse primers, which were intended to cover all six functional alpaca immunoglobulin heavy-chain joining (IGHJ) germline genes and, together with the forward primers, to reconstruct the entire VH/VHH transcript (FR1–FR4). Four reverse primers were designed as a common set shared by both the P3 and P4 primer sets (Supplementary Table S1). All forward and reverse primers carried a 5′ extension containing an SfiI recognition site, allowing directional cloning of the amplified VH/VHH fragments into the pComb3XSS phagemid vector (Creative Biogene, Shirley, NY, USA; Cat. No. VPT4013). Consequently, 40 (10 forward × 4 reverse) and 28 (7 forward × 4 reverse) independent primer pairs were available for the P3 and P4 libraries, respectively.

### 2.5. High-Throughput Sequencing (HTS) Analysis for the Selection of P3 Forward Primers

To determine which of the 32 possible forward primer sequences were predominantly utilized during amplification, alpaca VHH repertoires were amplified with the degenerate forward primer described above and sequenced on an Illumina NovaSeq 6000 platform (Illumina, San Diego, CA, USA) in 2 × 250 bp paired-end mode. A total of 37 repertoire datasets were analyzed, which were derived from PBMC samples collected from alpacas maintained under the same IACUC-approved protocol as described above (Approval No. SNU-240930-1; Approval No. SNU-240930-1-1).

Raw sequencing reads were processed as described previously [36], except that the primer regions were retained rather than trimmed, and the processed sequences were used to calculate the proportion of final sequences amplified by each forward primer. For each unique sequence, the region corresponding to the annotated forward primer length was extracted from the 5′ end and compared with the 32 reference primer sequences. A given VHH transcript was considered to have been amplified by a particular forward primer when the extracted region aligned to that primer with three or fewer mismatches, and sequences that could not be assigned to any reference primer under this criterion were excluded from further analysis.

To ensure that the selected primers would capture both the diversity and the abundance structure of the VHH repertoire, the abundance of each primer was quantified at two levels: the number of unique sequences assigned to that primer, reflecting the breadth of the repertoire it covers, and the total number of reads associated with those sequences, reflecting the contribution of that primer to highly abundant transcripts. Both values were expressed as fractions of the total number of unique sequences and of the total read count within each dataset, respectively, allowing primer usage to be compared across datasets. Because a primer contributing only a small number of unique sequences may nonetheless account for a substantial proportion of the transcripts actually present in the repertoire, both metrics were considered jointly, and primers showing consistent and substantial usage in either measure across the 37 datasets were selected as the P3 forward primer set, resulting in the selection of 10 of the 32 possible sequences (Supplementary Table S1).

### 2.6. Generation of VH and VHH Alpaca Phage Display Library

Alpaca phage display libraries were constructed using total RNA prepared from the 50 mL of PB sample as described previously [25]. Briefly, total RNA was used to synthesize complementary DNA (cDNA) using SuperScript™ IV Reverse Transcriptase (Invitrogen, Carlsbad, CA, USA; Cat. No. 18090200) and oligo(dT)20 primer (Invitrogen, Carlsbad, CA, USA; Cat. No. 18418020). The cDNA was used to amplify the VH/VHH gene fragments using the P3 (VHH) and P4 (VH/VHH) primer sets (Supplementary Figure S1) as described above and KAPA HiFi HotStart PCR Kit with dNTPs (KAPA Biosystems, Wilmington, MA, USA; Cat. No. KK2502). The PCR conditions were as follows: 95 °C for 5 min; 25 cycles of 98 °C for 30 s, 64 °C for 45 s, 72 °C for 1 min; and 72 °C for 7 min. The amplified VH/VHH gene fragments were then subjected to electrophoresis on a 1.5% agarose gel (LaboPass™ Agarose; Cosmo Genetech, Seoul, Republic of Korea; Cat. No. COC1001-051) and purified. During gel extraction, the bands corresponding to products amplified using the P3 primer pairs were pooled (Supplementary Figure S1a) and those amplified using the P4 primer pairs were pooled (Supplementary Figure S1b), respectively. The pooled products were purified using the NucleoSpin Gel and PCR Clean-up Kit (MACHEREY-NAGEL, Düren, Germany; Cat. No. 740609.50), following the manufacturer’s instructions.

The purified VHH (P3) and VH/VHH (P4) gene fragments were digested with SfiI restriction endonuclease (New England Biolabs, Ipswich, MA, USA; Cat. No. R0123S) and cloned into a pComb3XSS phagemid vector (Creative Biogene, Shirley, NY, USA; Cat. No. VPT4013) as described previously [43]. Then, *E. coli* K-12 ER2738 cells (New England Biolabs, Ipswich, MA, USA; Cat. No. E4104) were transformed with the phagemid vectors by electroporation and allowed to recover for 1 h at 37 °C with shaking at 220 rpm. After adding 10 mL of prewarmed (37 °C) Super Broth (SB; 30 g/L tryptone (US Biological, Salem, MA, USA; Cat. No. T8750), 20 g/L yeast extract (Glentham Life Sciences, Corsham, UK; Cat. No. GE4420), 10 g/L 3-(N-morpholino)propanesulfonic acid [MOPS] (Glentham Life Sciences, Corsham, UK; Cat. No. GB3672), and 3 µL of 100 mg/mL carbenicillin disodium salt (Gold Biotechnology, St. Louis, MO, USA; Cat. No. C-103-50) to the culture, the transformed bacteria were titrated by diluting 2 µL of the culture in 200 µL of SB medium and plating 100 and 10 µL of this 1:100 dilution onto separate Luria–Bertani (LB) agar plates (LB Broth Miller and bacteriological agar, both from Formedium, Swaffham, UK; Cat. No. LMM012 and AGR10, respectively) containing 100 μg/mL carbenicillin disodium salt (LBC agar plates). Library complexity (in colony-forming units, CFU) was determined from the number of independent transformants counted the next day, as described previously [43].

The culture was incubated for an additional 1 h at 37 °C with shaking at 220 rpm, after which carbenicillin was added to a final concentration of 50 μg/mL. VCSM13 interference-resistant helper phage (Agilent Technologies, Santa Clara, CA, USA; Cat. No. 200251) was then added to the culture (2 mL of a 4 × 10^12^ PFU/mL) as described previously [44], and the culture was incubated for 1.5–2 h at 37 °C with shaking at 220 rpm to allow phage infection. Kanamycin monosulfate (Gold Biotechnology, St. Louis, MO, USA; Cat. No. K-120-25) was subsequently added to a final concentration of 70 μg/mL to select for helper phage–infected cells, and the culture was incubated overnight at 37 °C with shaking at 220 rpm to produce phage particles as described previously [43]. Phage particles were then precipitated from the culture supernatant using polyethylene glycol 8000 (PEG-8000) (Sigma-Aldrich, St. Louis, MO, USA; Cat. No. 81268) and sodium chloride (Sigma-Aldrich, St. Louis, MO, USA; Cat. No. S9888) as described previously [43], and the resulting fresh phage preparation was either used for the next round of bio-panning or stored as described previously [36,43].

### 2.7. Bio-Panning and Sequencing of Colonies

The P3 (VHH) and P4 (VH/VHH) libraries were subjected to three rounds of bio-panning against recombinant 27-60 scFv-hFc-HA fusion protein as described previously [36,40,43,45]. Briefly, for every round of bio-panning, the antigen-coated beads (3 µg of antigen in 5.0 × 10^6^ Dynabeads™ M-270 Epoxy [Invitrogen, Carlsbad, CA, USA; Cat. No. 14301]) were incubated with each phage library containing approximately 10^12^–10^13^ infectious phages for 2 h at 37 °C, except for the first round, which used twice the number of antigen-coated beads (6 µg of antigen in 1.0 × 10^7^ Dynabeads™ M-270 Epoxy). The number of washes was gradually increased across rounds: a single wash with 500 μL of PBS containing 0.05% (*v*/*v*) TWEEN^®^ 20 (PBST; Sigma-Aldrich, St. Louis, MO, USA; Cat. No. P1379-500ML) in the first round, followed by three and five washes in the second and third rounds, respectively. After each round of bio-panning, the bound phages were eluted and amplified for the next round, as described previously [36,40]. Input and output infectious phage titers were determined at each round by infecting *E. coli* K-12 ER2738 with serial dilutions of the phage preparation and counting carbenicillin-resistant colonies (Supplementary Table S2).

**Figure 1:**
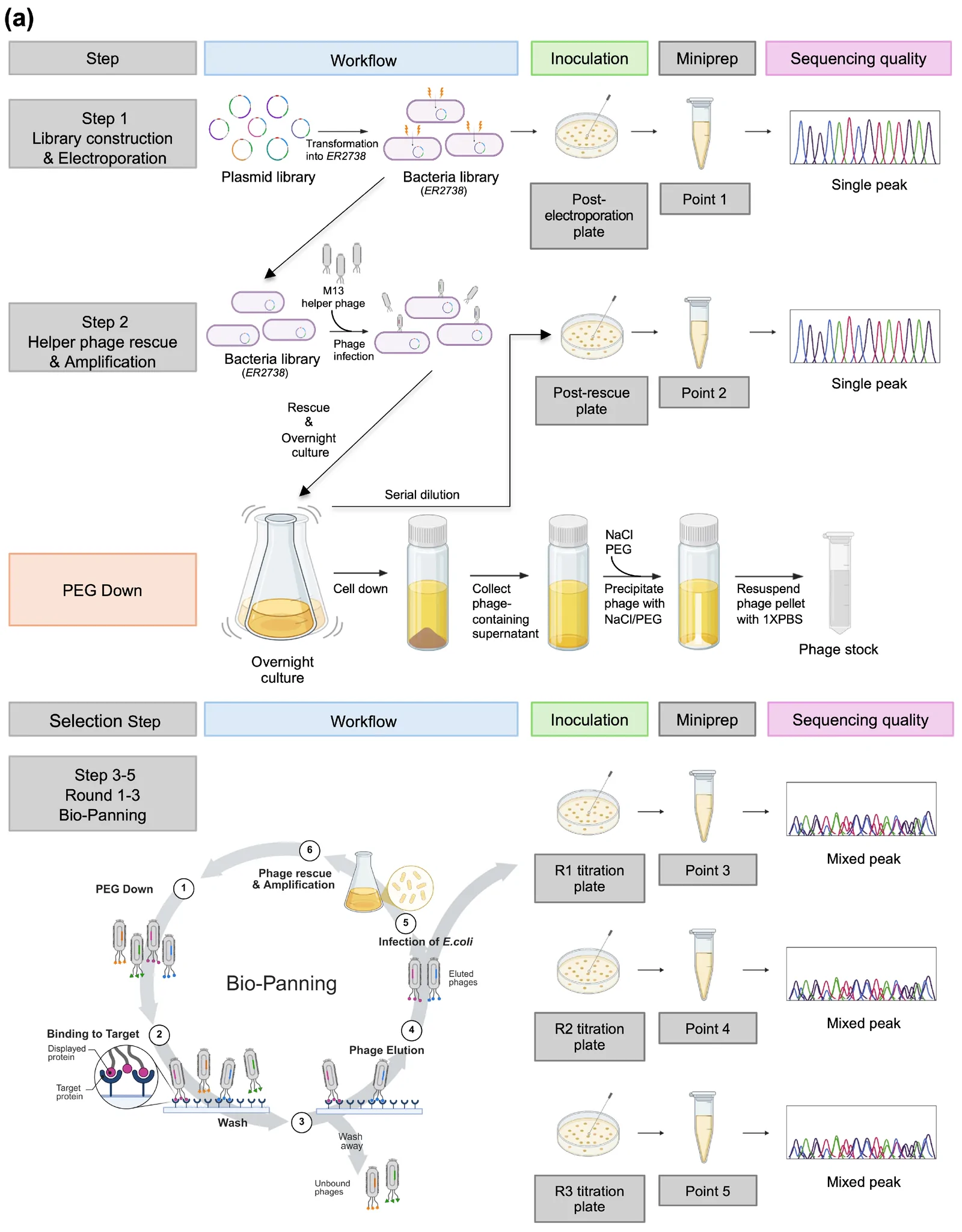

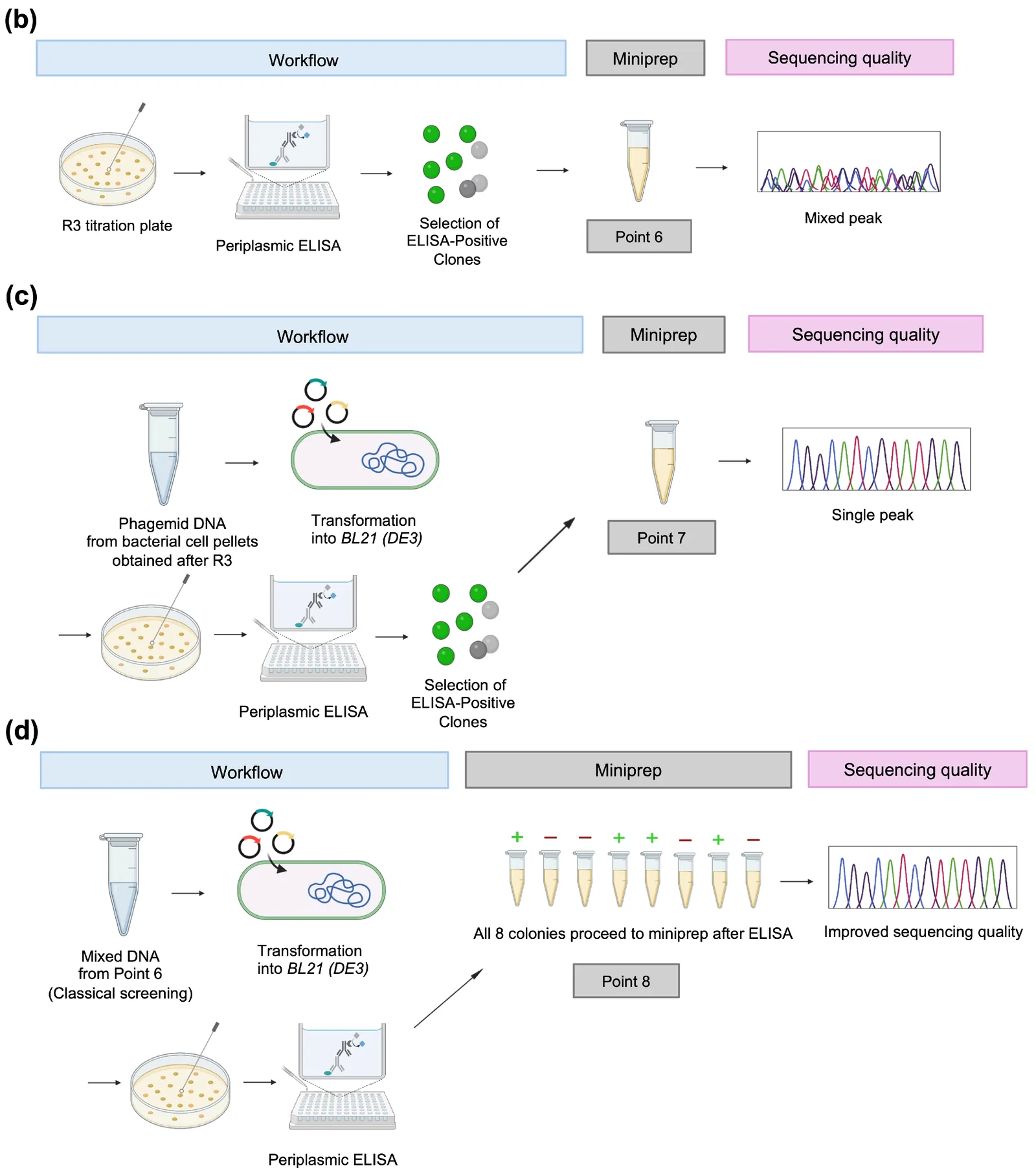
Workflow of alpaca phage display library construction, bio-panning, plasmid DNA preparation, and sequencing. (**a**) Schematic representation of the experimental workflow illustrating phage display library construction and bio-panning. Sequencing was performed at five points, using colonies from the titration plates of the vector transformation (Point 1), the plate obtained by serial dilution of the overnight rescue culture after helper phage infection (Point 2), and the output titration plates of three consecutive bio-panning rounds (Points 3–5). (**b**) Schematic illustration of the classical screening strategy. Individual colonies were randomly picked directly from the output titration plates of the third bio-panning round. Periplasmic extracts containing VH/VHH antibodies were subjected to ELISA using microtiter plates coated with recombinant 43-09 scFv-hFc. The corresponding ELISA results are presented in Supplementary Figure S2. ELISA-positive clones were subjected to Sanger sequencing (Point 6). (**c**) Schematic illustration of the optimized screening strategy. Following the overnight culture after the third round of bio-panning, phagemid DNA was prepared from the bacterial cell pellet and transformed into naïve Escherichia coli BL21(DE3) cells, which were then plated. From the resulting colonies, periplasmic extracts containing VH/VHH antibodies were prepared and subjected to ELISA using microtiter plates coated with recombinant 43-09 scFv-hFc. The corresponding ELISA results are presented in Supplementary Figure S3. ELISA-positive clones were subjected to Sanger sequencing (Point 7). (**d**) Five ELISA-positive clones exhibiting mixed chromatograms, as identified by classical screening, were selected from each of the P3 and P4 libraries for further analysis (P3-19, P3-38, P3-44, P3-49, and P3-83 from the P3 library; P4-13, P4-26, P4-33, P4-79, and P4-83 from the P4 library). Their phagemid DNA was retransformed into E. coli BL21(DE3) cells, and transformants were selected on LB agar plates supplemented with carbenicillin. Eight colonies were randomly picked from each plate and used to prepare periplasmic extracts, which were analyzed for antigen reactivity by ELISA using microtiter plates coated with recombinant 43-09 scFv-hFc. The corresponding ELISA results are presented in Supplementary Figure S4. All clones were subjected to Sanger sequencing (Point 8). Created in BioRender. Chung, J. (2026) https://BioRender.com/qrt3e9c, accessed on 22 September 2026.

For Sanger sequencing, phagemid DNA samples were prepared from bacterial colonies throughout the initially transformed library, helper phage–rescued library, and bio-panning: Points 1–5 (Figure 1a). Two independent alpaca antibody libraries, designated P3 (VHH) and P4 (VH/VHH), were analyzed throughout the study. At each step, twenty colonies were randomly selected from each library and subjected to plasmid extraction using the GenonPrep™ Plasmid Mini Kit (GenonLAB, Seoul, Republic of Korea; Cat. No. GP-PS1030) according to the manufacturer’s instructions. Sanger sequencing was performed by Bionics Co., Ltd. (Seoul, Republic of Korea).

At Point 1, following the electroporation of P3 and P4 libraries into *E. coli* K-12 ER2738 cells, the transformed bacteria were titrated by diluting 100-fold using SB medium and plating 100 and 10 µL of this diluted culture onto separate LBC agar plates. After overnight incubation at 37 °C, twenty colonies were randomly selected from the titration plates of each library. After infection with VCSM13 helper phage and overnight phage amplification [40], a portion of the rescued culture was serially diluted 10^4^-fold, and 100 µL of this diluted culture was plated onto separate LBC agar plates. From each plate, twenty colonies from each library were randomly selected (Point 2). Twenty colonies were also selected from the output titration plates of the first, second, and third rounds of bio-panning for each of the P3 and P4 libraries (Points 3, 4, and 5) [40].

Sanger sequencing chromatograms were classified using manual inspection of electropherogram morphology together with a quantitative base-quality criterion. Chromatograms were first assessed for interpretability across the predefined VH/VHH region, extending from FR1 to the end of the C-terminal histidine (His) tag. Reads with globally weak, noisy, or incomplete signal; without a consistent peak register; or without an identifiable VH/VHH sequence spanning this window were classified as sequencing failures. These reads could not be trimmed to the predefined analysis window and were excluded before quality-based classification.

Interpretable chromatograms were trimmed from FR1 to the end of the His tag. The percentage of high-quality bases (%HQ) within the trimmed region was calculated using Geneious Prime^®^ version 2025.2.2 (Biomatters Ltd., Auckland, New Zealand) [46]. Base calls were classified using the default Geneious quality-binning profile as low quality (LQ; Q < 20), medium quality (MQ; Q20–39), or high quality (HQ; Q ≥ 40). Thus, %HQ represented the proportion of bases with Q ≥ 40; Q40 corresponds to an estimated per-base error probability of 1.0 × 10^−4^ [47,48].

Interpretable chromatograms with a clear single-peak profile throughout the trimmed region and %HQ ≥ 95% were classified as single-peak sequences. Chromatograms with resolvable overlapping or multiple peaks at one or more positions and %HQ < 95% were classified as mixed-peak sequences (Supplementary Figure S5). Visual morphology and %HQ were assessed independently and were concordant across the dataset. The distribution of %HQ values showed an abrupt decrease in the number of reads immediately below 95% (Supplementary Figure S6). This separation supported the use of 95% as the %HQ cutoff, together with electropherogram morphology, for classifying single- and mixed-peak sequences.

### 2.8. Classical Screening of Antigen-Binding Clones

From the Round 3 output titration plates (Figure 1b), individual colonies were inoculated into 1 mL of SB medium containing 100 μg/mL carbenicillin (SBC) in Axygen^®^ 96-well 2.2-mL polypropylene deep-well plates (Corning, Corning, NY, USA; Cat. No. P-2ML-SQ-C) and cultured overnight at 37 °C with shaking at 220 rpm. Subsequently, 50 µL of the overnight culture was transferred into 850 µL of fresh SBC medium and incubated at 30 °C with shaking at 220 rpm until the optical density at 600 nm (OD600) reached 0.7. 

Then, isopropyl β-D-1-thiogalactopyranoside (IPTG) (Gold Biotechnology, St. Louis, MO, USA; Cat. No. I2481C) was added to a final concentration of 1 mM to induce the expression of VH/VHH fragments, and the culture was then incubated overnight at 18 °C with shaking at 220 rpm as described previously [49]. Bacterial cell pellets were obtained by centrifugation at 5000× *g* for 20 min at 4 °C, resuspended in 120 µL of TES buffer (50 mM Tris-HCl, pH 8.0, 20% sucrose (Sigma-Aldrich, St. Louis, MO, USA; Cat. No. 84097), 1 mM ethylenediaminetetraacetic acid [EDTA] (Sigma-Aldrich, St. Louis, MO, USA; Cat. No. 03677)), and incubated on ice for 30 min. Subsequently, 180 µL of an ice-cold solution comprising 177 μL of water and 3 μL of 100 mM PMSF (phenylmethylsulfonyl fluoride; Sigma-Aldrich, St. Louis, MO, USA; Cat. No. P7626-25G) stock solution (final PMSF concentration, 1 mM) was added, and the samples were incubated on ice for an additional 30 min. After centrifugation at 5000× *g* for 15 min, the supernatants containing periplasmic proteins were collected for ELISA.

To assess whether mixed-peak chromatograms resulted from the accidental co-picking of adjacent bacterial colonies, two antigen-binding clones that had yielded mixed-peak chromatograms (P3-47 and P3-65) were selected from the P3 library and streaked onto LBC agar plates to obtain well-isolated single colonies, yielding 96 individual colonies per clone. These colonies were processed according to the classical screening procedure described above and in Section 2.9 (Supplementary Figure S7a), and the antigen-binding clones identified were subjected to plasmid preparation and Sanger sequencing.

### 2.9. Periplasmic ELISA

Wells of Corning^®^ 96-well half-area high-binding microplates (Corning, Corning, NY, USA; Cat. No. 3690) were coated with 100 ng of recombinant 43-09 scFv-hFc fusion protein in 20 µL of coating buffer (0.1 M sodium bicarbonate buffer, pH 8.3, prepared in house from sodium bicarbonate (Sigma-Aldrich, St. Louis, MO, USA; Cat. No. S5761)) at 4 °C overnight. The 43-09 scFv-hFc protein contains a scFv region distinct from that of the 27-60 scFv-hFc-HA protein used for immunization and bio-panning, while retaining the same hFc region. It was therefore used as the screening antigen to preferentially identify clones recognizing the shared hFc component rather than epitopes unique to the 27-60 scFv or HA tag.

The wells were then blocked with 150 µL of 3% (*w*/*v*) Probumin^®^ Bovine Serum Albumin (BSA) (MilliporeSigma, Burlington, MA, USA; Cat. No. 821006-100G) in PBS for 1 h at 37 °C as described previously, with minor modifications [25]. Periplasmic extracts containing expressed VH/VHH proteins were added to the antigen-coated wells (50 µL/well). After incubation for 2 h at 37 °C and washing three times with PBST, anti-HA–horseradish peroxidase (HRP) high-affinity antibody (clone 3F10; Roche Diagnostics, Mannheim, Germany; Cat. No. 12013819001; diluted 1000-fold in blocking buffer) was added (50 µL/well). The plate was incubated for 1 h at 37 °C and washed three times with PBST. Then, 2,2′-azino-bis(3-ethylbenzothiazoline-6-sulfonic acid) (ABTS) solution (Invitrogen, Carlsbad, CA, USA; Cat. No. 002024) was added (50 µL/well) as an HRP substrate. After incubation for 15 min at 37 °C, the absorbance was measured at 405 nm using a Multiskan™ GO microplate spectrophotometer (Thermo Fisher Scientific, Vantaa, Finland). To determine the background signal, BSA-blocked wells were used.

To assess the periplasmic expression level of VH/VHH per clone, wells were coated directly with 50 µL of periplasmic extract (without prior antigen coating) for 1 h at 37 °C, and then incubated with anti-HA-HRP conjugate (1:1000 in blocking buffer) for 1 h at 37 °C. The reaction was developed and measured as described above, with the resulting signal representing the relative expression level of each clone.

Clones were considered positive when the absorbance at 405 nm (OD405) was ≥1.5 in classical screening and ≥1.0 in optimized screening. These cutoffs were selected empirically after examination of the experimental OD405 distributions shown in Supplementary Figures S2 and S3.

### 2.10. Optimized Screening of Antigen-Binding Clones

After the third round of bio-panning and overnight culture, the culture was centrifuged at 4000× *g* for 10 min at 4 °C. Phagemid DNA was prepared from the cell pellet using the PureLink™ HiPure Plasmid Midiprep Kit (Invitrogen, Thermo Fisher Scientific, Waltham, MA, USA; Cat. No. K210004) according to the manufacturer’s instructions. The phagemid DNA samples were transformed into *E. coli* BL21(DE3) chemically competent cells (Sigma-Aldrich, St. Louis, MO, USA; Cat. No. CMC0014-4X40UL) and spread on an LBC agar plate (Figure 1c). Individual colonies were inoculated into 1 mL of SBC, and the remaining steps were performed identically to those described in the classical screening of antigen-binding clones. After the supernatants containing periplasmic proteins were collected, periplasmic ELISA was conducted as described above.

### 2.11. Retransformation of Phagemid DNA and Preparation of Periplasmic Extracts from Colonies

To resolve the mixed-peak chromatograms identified during classical screening, plasmid DNA individually prepared at Point 6 (Figure 1b) from five representative mixed-peak clones selected from each of the P3 and P4 libraries was retransformed separately into *E. coli* BL21(DE3) chemically competent cells, and each transformation was spread on a separate LBC agar plate (Figure 1d). Eight colonies were randomly selected from each retransformation plate, yielding a total of 80 colonies. Individual colonies were inoculated into 1 mL of SBC, and the remaining steps were performed identically to those described in the classical screening strategy of antigen-binding clones. After the supernatants containing periplasmic proteins were collected, periplasmic ELISA was conducted as described above. Regardless of the ELISA result, phagemid DNA was prepared from all 80 colonies and subjected to Sanger sequencing to determine whether retransformation resolved the mixed chromatograms into single sequences.

### 2.12. HCDR3 Alignment by the IMGT Numbering Scheme

To compare heavy-chain complementarity-determining region 3 (HCDR3) length and sequence diversity across selected antigen-binding clones, heavy-chain amino acid sequences were analyzed using the IMGT/V-QUEST tool (version 3.8.2; Montpellier, France) [50,51] to assign IMGT numbering and define HCDR3 boundaries (IMGT positions 105–117). The resulting HCDR3 sequences were then manually aligned according to IMGT positions.

### 2.13. Sequence-Based Classification of VH and VHH Clones

For VH/VHH classification, the Sanger sequencing result of each antigen-binding clone was translated in the correct reading frame, and the translated V-domain sequence was analyzed using ANARCI [52] (SAbPred web server; https://opig.stats.ox.ac.uk/webapps/sabdab-sabpred/sabpred/anarci/, accessed on 5 August 2026) to assign Kabat-numbered residues and the closest alpaca IGHV and IGHJ germline genes.

Clones were classified as VH or VHH according to the criteria described by Harmsen and De Haard [53], who identified substitutions at four FR2 positions—Kabat residues 37, 44, 45, and 47—as the most reliable distinguishing feature between the two antibody types. The expected VHH-type residue at each hallmark position was defined as phenylalanine or tyrosine at position 37, glutamate at position 44, arginine at position 45, and glycine at position 47. Each clone was scored according to the number of legible hallmark positions matching the expected VHH-type residue, and assigned a preliminary call of VHH (3–4/4 match), indeterminate (IND, 2/4), or VH (0–1/4).

Three additional sequence-based features described in the same report [53] were evaluated as supplementary evidence and applied only to resolve IND clones. First, the residue at Kabat position 103 was examined; this position is normally occupied by a conserved hydrophobic residue (typically tryptophan) that packs against the light-chain variable domain in VH, whereas a subset of VHHs instead carries a hydrophilic residue at this position. Second, HCDR3 length was measured as the number of residues between the conserved cysteine preceding FR4 and the start of the FR4 motif; HCDR3 loops of 20 residues or greater were considered VHH-consistent. No numerical threshold is specified in the source report [53], so this cutoff was used as a working approximation. Third, the total number of cysteine residues within the V-domain was counted, with more than two cysteines interpreted as evidence of an additional, non-canonical disulfide bond of the type associated with extended-HCDR3 VHHs. IND clones were reclassified as VHH if any one of the three supplementary criteria was met (hydrophilic residue at position 103, HCDR3 length ≥20 amino acid residues, or more than two cysteines in the V-domain); clones meeting none of these criteria remained classified as IND.

## 3. Results

### 3.1. Occurrence of Mixed Sequencing Chromatograms During Bio-Panning of P3 (VHH) and P4 (VH/VHH) Phage Display Libraries

To generate anti–human Fc sdAbs, we immunized and boosted one alpaca with a recombinant Fc fusion protein (27-60 scFv-hFc-HA) [36] and collected blood samples. Then, PBMCs were isolated and total RNA samples were prepared from the cells, from which cDNA was synthesized using an oligo(dT)20 primer. Gene fragments encoding the VHH and VH/VHH domains were amplified with the P3 and P4 primer sets from the cDNA, respectively, and subcloned into a phagemid vector (Supplementary Figure S1). The phagemid DNAs were transformed into *E. coli* to generate the P3 (VHH) and P4 (VH/VHH) phage display libraries, whose complexities were determined to be 3.33 × 10^8^ and 2.09 × 10^8^ CFU, respectively.

The rescued phages were subjected to the first round of bio-panning. The input and output infectious phage titers for each round are summarized in Supplementary Table S2. To monitor the occurrence of mixed sequencing chromatograms during library construction and bio-panning, twenty colonies per library were analyzed by Sanger sequencing at five sampling points (Points 1–5; Figure 1a), and the resulting chromatograms were classified as single-peak, mixed-peak, or sequencing failure (Supplementary Figure S5). 

**Figure 2:**
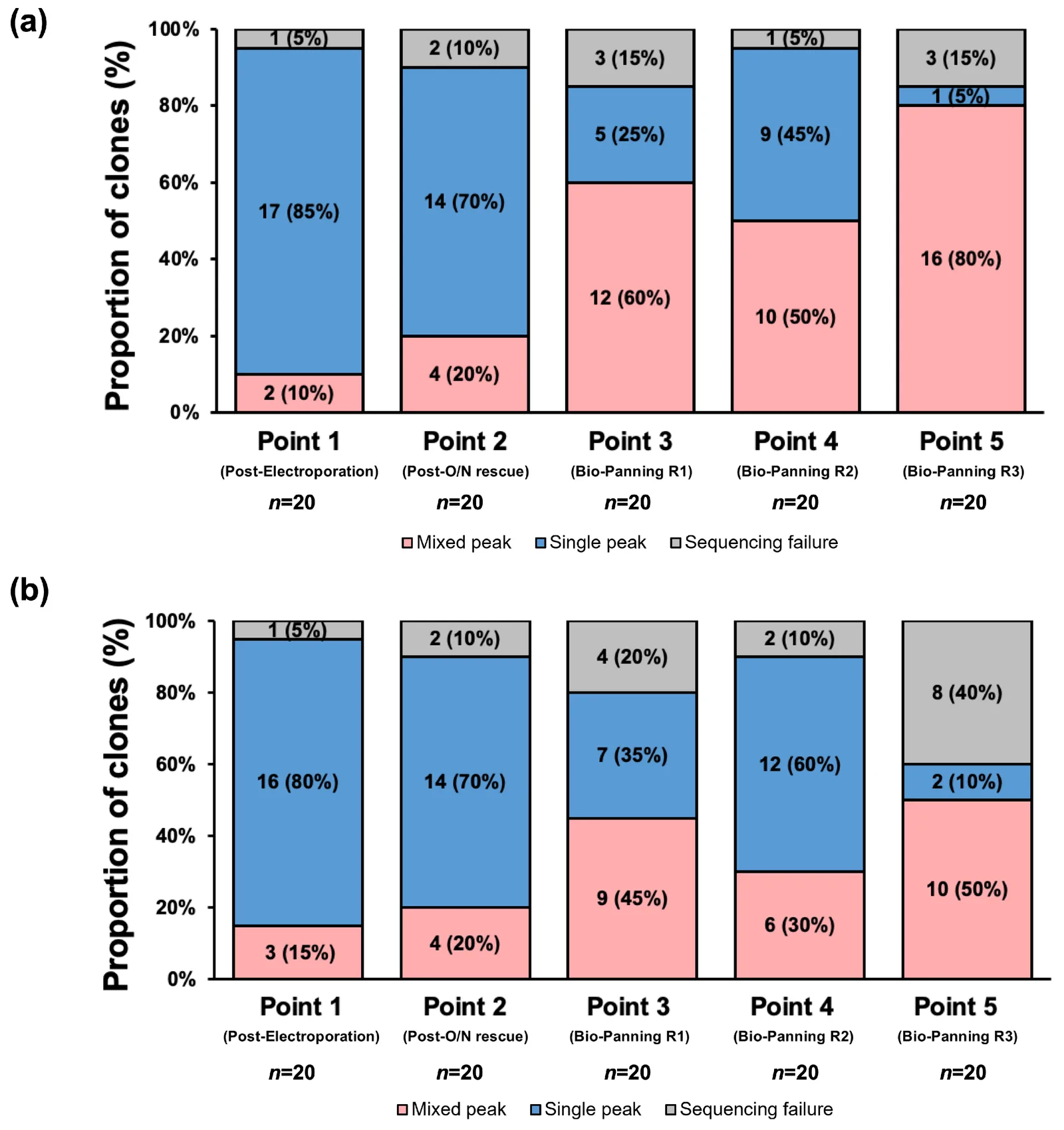
Occurrence of mixed Sanger sequencing chromatograms during construction and bio-panning of alpaca P3 (VHH) and P4 (VH/VHH) phage display libraries. Monitoring sequence quality throughout bio-panning of the (**a**) VHH (P3) and (**b**) VH/VHH (P4) phage display libraries. Twenty individual colonies were randomly selected at each point. Phagemid DNA was prepared and subjected to Sanger sequencing. Sequencing chromatograms were classified as mixed peak (red), single peak (blue), or sequencing failure (gray) based on the sequence quality classification criteria described in Supplementary Figure S5. Detailed data corresponding to this analysis can be found in Supplementary Table S3.

For the P3 library, mixed chromatograms were observed in 2/20 colonies at Point 1 (10%), 4/20 at Point 2 (20%), 12/20 at Point 3 (60%), 10/20 at Point 4 (50%), and 16/20 at Point 5 (80%). For the P4 library, mixed chromatograms were observed in 3/20 colonies at Point 1 (15%), 4/20 at Point 2 (20%), 9/20 at Point 3 (45%), 6/20 at Point 4 (30%), and 10/20 at Point 5 (50%) (Figure 2, Supplementary Table S3).

### 3.2. Classical Single-Colony Screening After Bio-Panning Failed to Recover Sequence-Defined Antigen-Binding Clones

**Figure 3:**
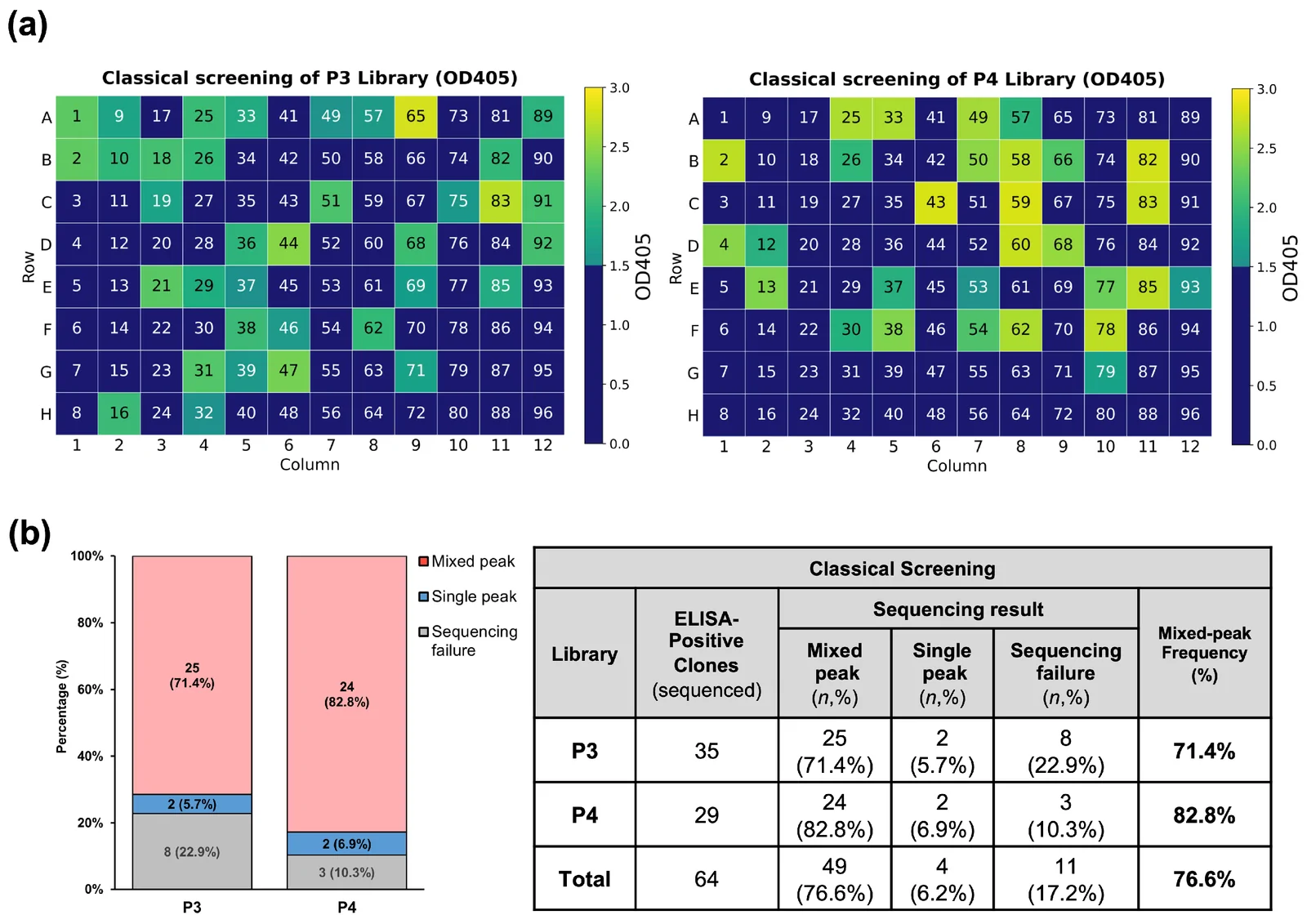
Classical screening of antigen-binding VH/VHH clones after bio-panning. (**a**) Heatmap representation of periplasmic ELISA results for 96 randomly selected clones from each of the P3 and P4 libraries. Color intensity represents the optical density (OD) at 405 nm. Clones showing OD < 1.5 at antigen-coated wells are shown in dark blue. (**b**) Sanger sequencing results of ELISA-positive clones. Sequencing chromatograms were categorized as mixed peak (red), single peak (blue), or sequencing failure (gray) based on the sequence-quality classification criteria described in Supplementary Figure S5.

After the third round of bio-panning, 96 colonies were randomly selected from each of the P3 and P4 libraries and subjected to periplasmic ELISA using a plate coated with a different recombinant human Fc fusion protein (43-09 scFv-hFc; Figure 1b). From the P3 and P4 libraries, 35 and 29 antigen-binding clones were selected, respectively (Figure 3a and Supplementary Figure S2). These antigen-binding clones were subsequently subjected to plasmid preparation for Sanger sequencing analysis.

Unexpectedly, most of the antigen-binding clones exhibited mixed sequencing chromatograms despite originating from apparently isolated single bacterial colonies. Specifically, among the 35 antigen-binding clones identified in the P3 library, only two clones (5.7%) produced interpretable single-peak chromatograms, whereas 25 clones (71.4%) exhibited mixed sequencing profiles (Figure 3b). Similarly, only two of the 29 antigen-binding clones (6.9%) from the P4 library generated single-peak chromatograms, while 24 clones (82.8%) showed mixed sequencing patterns (Figure 3b). Representative chromatograms demonstrated extensive peak overlap throughout the VH and VHH gene regions (Supplementary Figure S5), indicating the presence of heterogeneous phagemid populations within single colonies. These results demonstrated that classical screening of antigen-binding clones—periplasmic ELISA of single colonies from output titration plates followed by Sanger sequencing—was substantially compromised in alpaca P3 (VHH) and P4 (VH/VHH) phage display libraries.

To determine whether the mixed sequencing chromatograms resulted from the accidental co-picking of adjacent bacterial colonies, two clones exhibiting both positive binding signals and mixed chromatograms (P3-47, P3-65) were randomly selected from the 25 mixed-chromatogram clones identified in the P3 library (Point 6; Figure 1b) and re-streaked to obtain well-isolated single colonies (Supplementary Figure S7a). From the colonies derived from P3-47 and P3-65, 10 and 20 antigen-binding clones were selected, respectively, and subjected to plasmid preparation and Sanger sequencing analysis (Point 6-1 and Point 6-2). 

However, mixed sequencing chromatograms persisted after repeated streaking: among the 10 antigen-binding clones derived from P3-47, only four (40%) produced interpretable single-peak chromatograms, five (50%) exhibited mixed chromatograms, and one (10%) failed sequencing (Supplementary Figure S7b). Similarly, among the 20 antigen-binding clones derived from P3-65, only one (5%) produced a single-peak chromatogram, sixteen (80%) exhibited mixed chromatograms, and three (15%) failed sequencing. These results demonstrated that conventional colony purification alone is insufficient to eliminate mixed sequencing profiles, suggesting that the coexistence of multiple phagemid templates was unlikely to be explained simply by incomplete colony isolation, and prompted the development of an alternative screening strategy based on retransformation of pooled phagemid DNA.

### 3.3. Retransformation of Pooled Phagemid DNA Enabled Identification of Antigen-Binding Clones by Restoring Clonality

To implement a retransformation-based strategy, phagemid DNA was prepared from the bacterial pellets obtained after the third round of bio-panning of the P3 and P4 libraries and then transformed into *E. coli* BL21(DE3) cells (Figure 1c). From each of the P3 and P4 libraries, 96 colonies were randomly selected and subjected to periplasmic ELISA. The VH and VHH gene sequences of antigen-binding clones were analyzed by Sanger sequencing (Point 7). 

**Figure 4:**
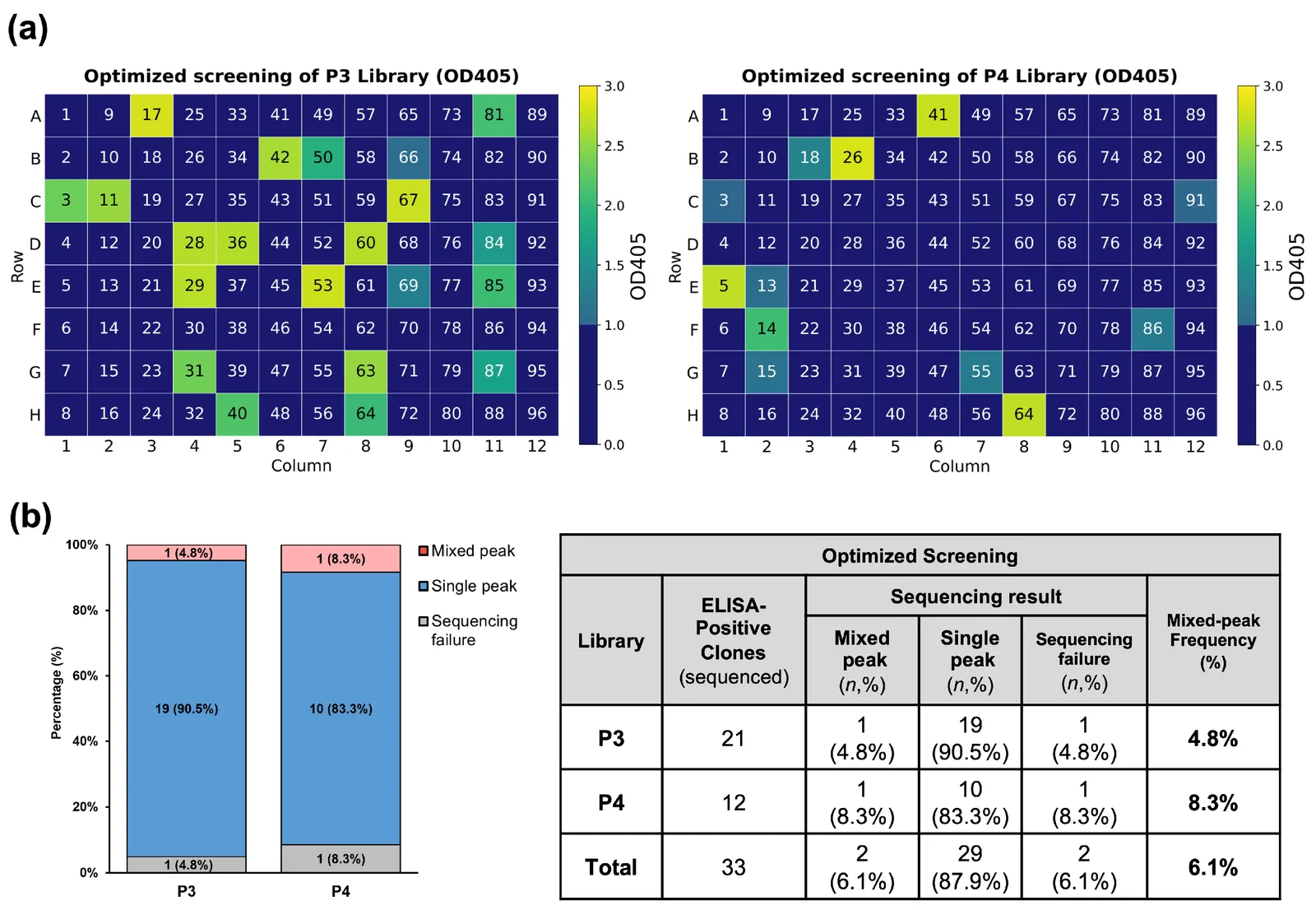

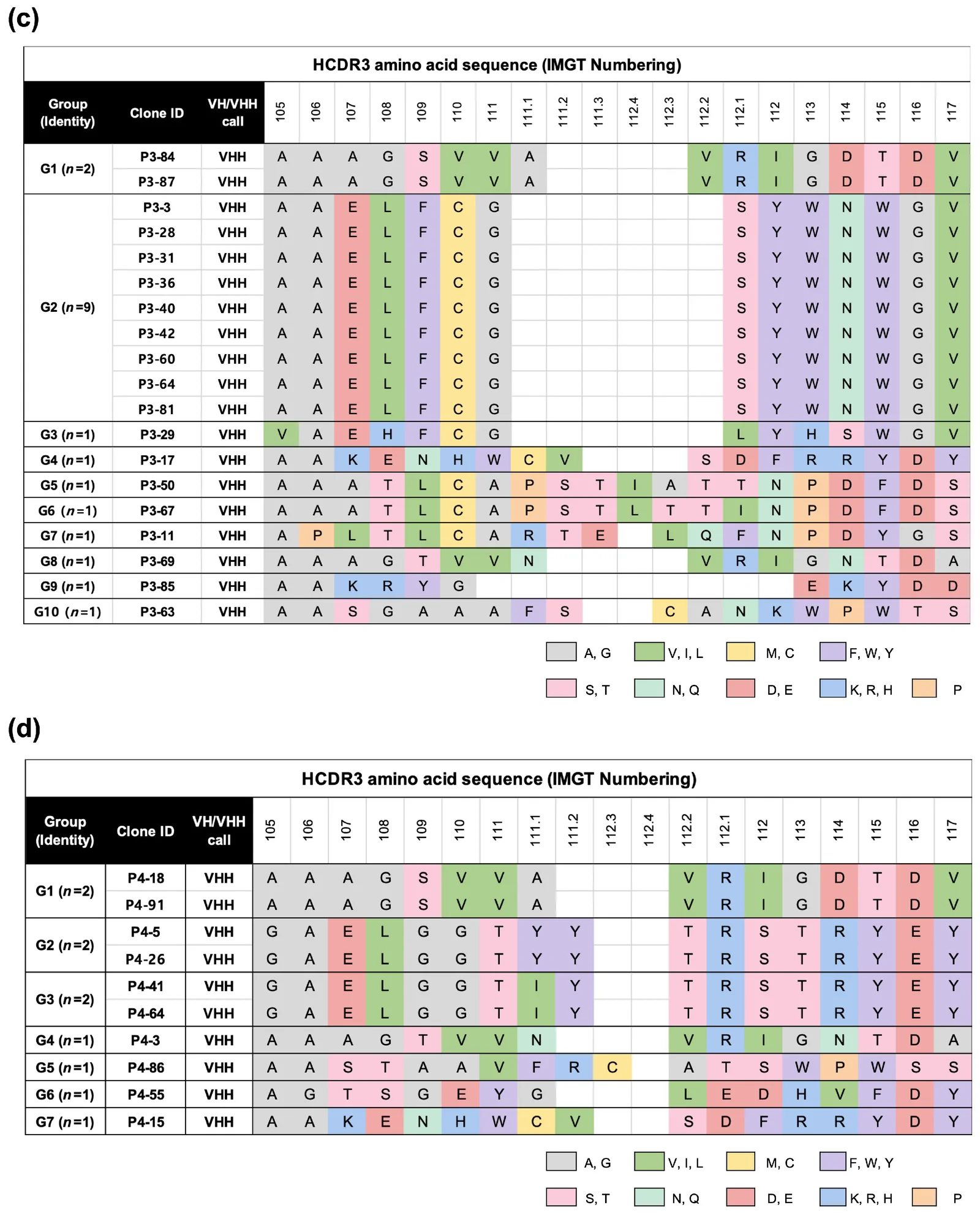
Optimized strategy for screening antigen-binding VH/VHH clones after bio-panning. (**a**) Heatmap representation of periplasmic ELISA results for 96 randomly selected clones from each of the P3 and P4 libraries. Color intensity represents the optical density (OD) at 405 nm. Clones showing OD < 1.0 at antigen-coated wells are shown in dark blue. (**b**) Sanger sequencing results of ELISA-positive clones. Sequencing chromatograms were categorized as mixed peak (red), single peak (blue), or sequencing failure (gray) based on the sequence-quality classification criteria described in Supplementary Figure S5. Percentages may not sum to 100% owing to rounding. (**c**,**d**) HCDR3 amino acid sequences of 19 and 10 single-peak antigen-binding clones from the P3 and P4 phage display libraries, respectively, were aligned according to the IMGT numbering scheme. VH/VHH classification was based on the four FR2 hallmark residues (Kabat positions 37, 44, 45, and 47); see Methods for details. These antigen-binding clones were identified by periplasmic ELISA, and the corresponding ELISA results are presented in Supplementary Figure S3. Clones sharing identical HCDR3 sequences were grouped together. Amino acids are color-coded according to their physicochemical properties.

Among the 21 antigen-binding clones from the P3 library, 19 clones (90.5%) produced single-peak chromatograms, and 10 of 12 antigen-binding clones (83.3%) from the P4 library also yielded single-peak chromatograms (Figure 4a,b, and Supplementary Figure S3). Overall, 29 of 33 antigen-binding clones (87.9%) yielded interpretable single-peak sequencing chromatograms, while two clones (6.1%) exhibited mixed sequencing profiles and two clones (6.1%) resulted in sequencing failure based on the sequence classification criteria (Supplementary Figure S5). Ultimately, Sanger sequencing identified 10 unique VHH sequences from 21 antigen-binding clones in the P3 library (Figure 4c). Notably, all 7 unique antibody sequences identified from 12 antigen-binding clones in the P4 library were also confirmed to be VHH (Figure 4d).

These results strongly suggested that the colonies in the output titration plates of the third round of bio-panning contained more than one phagemid clone, which can be separated into individual phagemid clones by retransforming pooled phagemid DNA into naïve *E. coli* cells.

### 3.4. Antigen-Binding Colonies Selected by Classical Screening Harbored Multiple Phagemids

In the classical screening, among 35 and 29 antigen-binding clones selected from the P3 and P4 libraries, 25 and 24 clones exhibited mixed-peak chromatograms (Point 6) (Figure 3b and Supplementary Figure S2), which suggested the presence of more than one phagemid clone within a single colony. To confirm this hypothesis, five representative clones showing mixed-peak chromatograms were randomly selected from each of the P3 (P3-19, P3-38, P3-44, P3-49, and P3-83) and P4 (P4-13, P4-26, P4-33, P4-79, and P4-83) libraries (10 clones in total), and their phagemid DNA was retransformed into naïve BL21(DE3) cells (Figure 1d). Eight colonies were randomly selected from each retransformation plate (a total of 80 colonies) and subjected to periplasmic ELISA (Supplementary Figure S4). The positive rates among the eight colonies derived from each original clone were variable, ranging from 0% to 50% for P3-derived colonies and 12.5% to 75% for P4-derived colonies. Overall, 8 of 40 colonies (20%) from the P3 library and 15 of 40 colonies (37.5%) from the P4 library showed positive signals in periplasmic ELISA. 

Regardless of ELISA results, all 80 colonies were subjected to phagemid DNA preparation and Sanger sequencing (Point 8; Figure 1d). Of the 80 colonies, 77 (96%) yielded single peaks—72 with intact sequences and 5 with frameshift mutations—whereas 1 gave a mixed peak and 2 failed sequencing.

**Figure 5:**
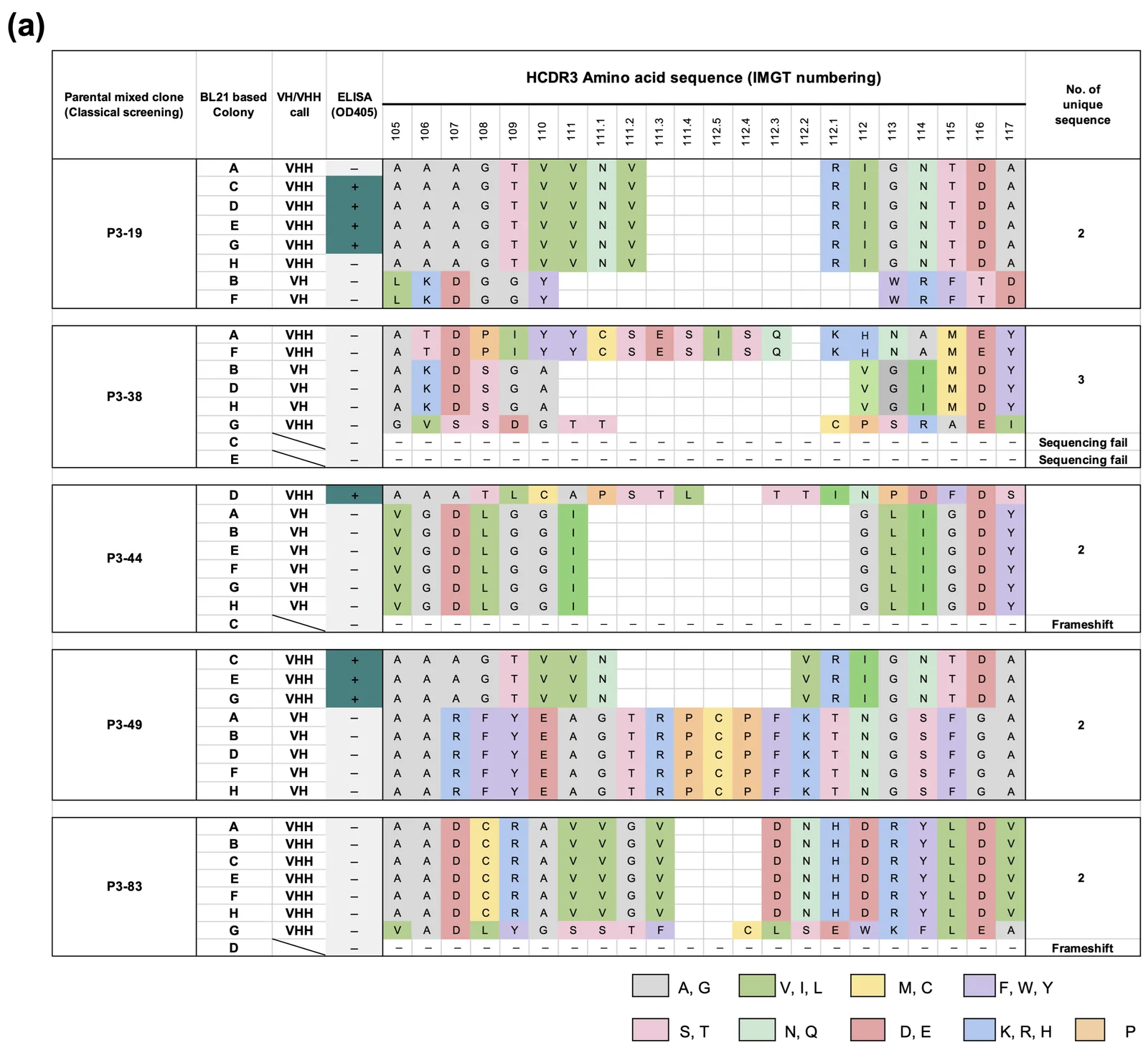

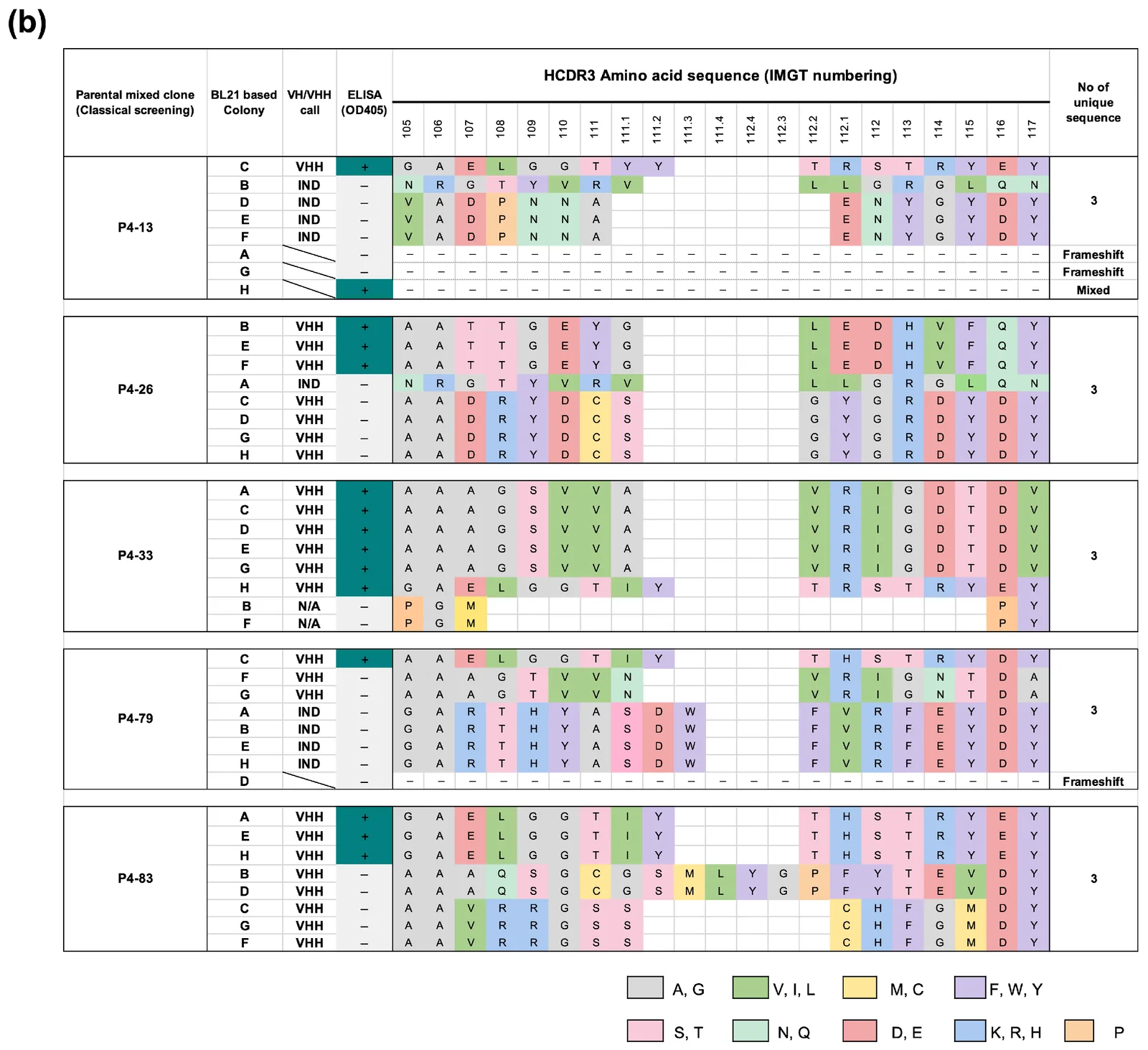
Retransformation of phagemid DNA prepared from mixed-peak colonies reveals multiple phagemids per colony. (**a**,**b**) HCDR3 amino acid sequences of 80 clones were aligned according to the IMGT numbering scheme. Sequencing chromatograms were categorized based on the sequence quality classification criteria described in Supplementary Figure S5. VH/VHH classification was based on the four FR2 hallmark residues (Kabat positions 37, 44, 45, and 47); see Methods for details. ELISA results were classified as positive (+) when the absorbance at 405 nm (OD405) was ≥1.0 and negative (−) when the OD405 was <1.0. The corresponding ELISA data are presented in Supplementary Figure S4. In the HCDR3 sequence alignments, hyphens (−) indicate positions for which no corresponding amino acid sequence was available due to frameshift, sequencing failure, or mixed peak. P4-33-B and P4-33-F each contain a premature ochre stop codon (TAA) in the FR2 region.

Each of the original 10 mixed-peak clones was found to comprise two to three distinct VHH or VH sequences (Figure 5), demonstrating in the analyzed clones that the mixed-peak chromatograms observed in the classical screening resulted from multiple phagemid clones coexisting within a single colony.

## 4. Discussion

Phage display remains one of the most widely used platforms for isolating antigen-specific antibodies, and its power rests on a strict genotype–phenotype linkage: the antibody displayed on each particle is physically tethered to the genome packaged inside it [1,2,38]. Here we describe a previously under-appreciated failure of this linkage at the colony level: a substantial fraction of the colonies recovered on the bio-panning output titration plate did not represent a single antibody clone, but instead harbored more than one phagemid clone in alpaca VH/VHH libraries.

The first indication of this was the recovery of mixed Sanger sequencing chromatograms from individual, well-isolated colonies. Such mixtures are usually attributed to the accidental co-picking of adjacent colonies carrying different phagemids. If that were the explanation, sub-cloning by re-streaking for single colonies should have separated the bacteria into genetically homogeneous clones, since streaking is the standard method for isolating a clonal population derived from a single progenitor cell [54,55,56]. Instead, colonies isolated after repeated streaking for single colonies continued to yield multiple sequencing peaks. This argues against incomplete colony isolation and instead indicates that multiple phagemid templates were maintained within the progeny of a single cell.

To test this directly, we prepared plasmid DNA from ten individual mixed-chromatogram colonies and transformed each preparation into naïve *E. coli*. The resulting colonies contained predominantly a single phagemid, and the original mixed chromatograms were thereby resolved into two to three distinct VH/VHH sequences (Figure 5). This confirms that the mixed chromatograms reflected the genuine coexistence of several phagemid templates within individual bacterial cells rather than a sequencing artifact, and that these templates can be physically separated once the phagemid population is re-introduced into naïve host cells by transformation.

Two routes could give rise to a single bacterial cell carrying multiple phagemids: (i) uptake of more than one phagemid molecule during the transformation used to build or amplify the library, or (ii) continued infection of a single cell by more than one phage particle during the bio-panning cycles that follow helper phage addition. The first route is well documented: *E. coli* can be simultaneously transformed by two or more plasmids—including plasmids that share the same selectable marker—and recent work indicates that such co-transformation is considerably more frequent than classically assumed, producing aclonal colonies whose plasmids are randomly reassigned among daughter cells in a DNA-concentration-dependent manner [57,58]. Nevertheless, our data favor the second route. When phagemid DNA was delivered into naïve cells by transformation under our conditions, the resulting colonies were overwhelmingly monoclonal. Had co-transformation been the dominant source, transformation would have been expected to reproduce the mixed populations, which it did not. In contrast, during bio-panning the host cells acquire phagemids exclusively by phage infection, which requires the F pilus and the TolA co-receptor [59]. Under the high phage titers generated during helper phage rescue and re-amplification, freshly divided daughter cells with F pili but already containing phagemid DNA can be reinfected by a different phage, especially before they express enough pIII to establish superinfection immunity, and thereby accumulate more than one phagemid [37]. Multiple infection during the amplification phase of bio-panning is therefore the more plausible origin of the multi-phagemid colonies we observed, although other mechanisms may also contribute to colony-level heterogeneity.

Notably, this phenomenon was pronounced for the VH/VHH phage display libraries but is not commonly reported for scFv- or Fab-based libraries. In the pComb3X phagemid display system used in this study, the antibody fragment forms a fusion protein with the C-terminal (anchor) domain of pIII, replacing the N-terminal N1 and N2 domains, which bind TolA and the F pilus to mediate infection [37,59]. An infective phagemid particle therefore relies on wild-type pIII derived from the helper phage; a displaying particle carries both the antibody fragment–pIII fusion and wild-type pIII, but its ability to infect *E. coli* depends on the wild-type pIII. Because wild-type pIII is provided in excess, standard helper phage rescue yields predominantly monovalent display, in which the fusion constitutes only a minority of the 3–5 pIII copies per particle—often fewer than one copy per particle—so that most particles carry only wild-type pIII even when they package the phagemid genome [37,60,61,62]. The N1 and N2 domains that engage TolA and the F pilus are thus supplied entirely by helper-derived wild-type pIII and are identical for scFv, Fab, and VH/VHH particles. Accordingly, the preferential accumulation of multiple phagemids in a single *E. coli* cell during VH/VHH phage display cannot be ascribed to the infection machinery itself. The format dependence might instead reflect how efficiently each construct is produced and how well the host grows during amplification. For a single cell to acquire two or more distinct phagemids, it must be co-infected by more than one phagemid-bearing particle during the overnight amplification, at a moment when it is still susceptible—that is, before it accumulates enough pIII to establish superinfection immunity. The frequency of such events is governed by two quantities: the titer, and hence the multiplicity of infection, of infective phagemid-bearing phage in the culture, and the supply of freshly divided, immunity-naïve daughter cells.

Single-domain antibodies might enhance both. A VH or VHH is a single 12–15 kDa domain that folds efficiently without V_H_–V_L_ pairing or an interchain disulfide. Its favorable folding and expression characteristics can promote efficient phage display [37,63] while imposing little metabolic or envelope-stress burden on the host. Consequently, VH/VHH rescue can yield a high titer of infective, phagemid-bearing particles, while the low growth toxicity permits vigorous division that continuously regenerates infection-susceptible daughters. The combination of a high multiplicity of infection and an ample supply of susceptible cells could make co-infection by more than one phagemid common. Larger, more complex formats could behave oppositely: scFv (~28 kDa) and especially Fab (~50 kDa, a two-chain, disulfide-linked assembly) fold and assemble inefficiently in the periplasm, load the Sec export pathway, and are prone to growth toxicity and to interference with pIII assembly from unpaired cysteines [64,65,66,67,68]. These liabilities could lower both the yield of infective phagemid-bearing phage and the host growth rate, so the multiplicity of infection stays low, and few susceptible daughters are generated; co-infection is correspondingly rare, and colonies remain clonal.

This phenomenon has direct practical consequences for antibody discovery. Because periplasmic ELISA measures antigen binding at the colony level [69], a positive signal from a colony that contains several phagemids cannot be clearly assigned to a single VH/VHH sequence. Genuine binders may thus go unidentified despite scoring ELISA-positive, and colony-level clonality is broken [2,38,70]. The limitation of classical colony-based screening therefore extends beyond insufficient sampling depth: even the colonies that are analyzed may not yield an interpretable, sequence-defined clone.

This problem can be addressed by two principal strategies. The first, described in the Results, is a retransformation approach: the pooled phagemid DNA recovered from the culture of the final round of bio-panning is transformed into naïve *E. coli* to generate a fresh set of colonies, which are then screened by periplasmic or phage ELISA, and antigen-binding clones are subjected to Sanger sequencing. Because transformation delivers predominantly one phagemid per cell, this restores clonality before screening, increasing the proportion of single-peak chromatograms from 6.2% to 87.9% (Figure 3b and Figure 4b), and thereby substantially improving the recovery of sequence-defined, antigen-binding clones. The second strategy is to bypass colony-based sequencing altogether by applying HTS to the selected repertoire, as in our previous work [71,72,73,74]. We regard these approaches as complementary: HTS reports sequence abundance, diversity, and enrichment across the entire selected population, whereas retransformation enables the physical recovery of individual, sequence-defined clones for functional validation. The two therefore address distinct limitations of the antibody discovery workflow rather than substituting for one another.

Although the P4 library was designed with an expanded primer set to capture conventional VH genes in addition to VHH genes, all antigen-binding clones recovered by bio-panning from both the P3 and P4 libraries were VHHs, and no conventional VH binders were identified in either library (Points 7 and 8; Figure 4c,d and Figure 5). A similar VHH-only outcome was reported from a mixed VH/VHH phage display library, in which five triclocarban-binding clones were all VHH/sdAbs rather than conventional VHs [75]. This observation may reflect favorable VHH properties—including VHH-specific FR2 residues and, in some clones, extended HCDR3 loops and additional intradomain disulfides—that support autonomous folding, solubility, and stability [53]. 

Several limitations should be acknowledged. Although our retransformation results are most consistent with the coexistence of multiple phagemids within single cells arising through multiple phage infection, we did not directly visualize multiple phagemids within an individual cell, and the precise frequency and molecular determinants of multiple infection remain to be defined; single-cell plasmid analysis and copy-number quantification will be needed to test this directly [76]. To test the proposed format-dependent mechanism specifically, future studies could directly compare the infectious titers (transducing units, TU/mL) of purified sdAb- versus scFv/Fab-displaying phage. Finally, the prevalence of this phenomenon may depend on library design, vector architecture, host strain, helper phage system, and bio-panning conditions [38], and whether comparable mixed chromatograms occur across other display platforms and antibody formats warrants further study. Clarifying these points will help establish more robust and reproducible phage display workflows.

## 5. Conclusions

In alpaca VH/VHH phage display libraries, an individual colony recovered after bio-panning does not reliably correspond to a single phagemid. Mixed-peak chromatograms persisted through repeated streaking for single colonies, and retransformation of phagemid DNA from such colonies into naïve *E. coli* separated each of the colonies examined into distinct VH/VHH sequences. Colony-level clonality, an assumption usually taken for granted in colony-based screening, is therefore not strictly preserved under these conditions. Multiple phage infection during the amplification phase of bio-panning, rather than co-transformation during library construction, is the more plausible origin of this heterogeneity, although the mechanism was not directly established.

The practical consequence is that classical single-colony screening yielded interpretable, sequence-defined clones from only a small minority of antigen-binding colonies in either library. After transformation of the pooled phagemid DNA, most such clones gave single-peak chromatograms. Inserting a retransformation step between the final round of bio-panning and screening is thus a simple way to restore clonality and to recover antigen-binding clones that would otherwise go unidentified.

## Data Availability

The raw sequencing data generated in this study have been deposited in the NCBI Sequence Read Archive (SRA) under BioProject accession number PRJNA1508170.

## Supplementary Materials

The following supporting information can be downloaded at: https://www.mdpi.com/article/10.3390/biom16101523/s1, Table S1. Sequences of primer sets used for amplification of the genes encoding alpaca VHH and VH domains. Figure S1. PCR amplification of gene fragments encoding alpaca VHH and VH domain using P3 and P4 primer sets. Table S2. Input and output infectious phage titers across three rounds of bio-panning for the P3 and P4 libraries. Figure S2. ELISA results of clones from the P3 and P4 phage display libraries. Figure S3. Periplasmic ELISA results of clones obtained by retransformation of phagemid DNA pools from the P3 and P4 phage display libraries after the third round of bio-panning. Figure S4. ELISA results of 80 clones obtained by retransformation of 10 antigen-binding clones selected by classical screening from the P3 and P4 phage display libraries. Figure S5. Representative Sanger sequencing chromatograms illustrating the sequence quality classification criteria. Figure S6. Distribution of %HQ values across single-peak, mixed-peak, and sequencing-failure chromatograms. Figure S7. Sequencing results of phagemid DNA from colonies formed by restreaking the P3-47 and P3-65 clones. Table S3. Sequencing results of the P3 and P4 phage display libraries at each point of study.

## Abbreviations

The following abbreviations are used in this manuscript:

ABTS	2,2′-azino-bis(3-ethylbenzothiazoline-6-sulfonic acid)
ANARCI	Antigen receptor Numbering And Receptor Classification (antibody numbering software)
aTTP	acquired thrombotic thrombocytopenic purpura
BSA	bovine serum albumin
cDNA	complementary DNA
CDR3	complementarity-determining region 3
CFU	colony-forming unit
C_H_1	constant domain 1 of the heavy chain
C_L_	constant domain of the light chain
DNA	deoxyribonucleic acid
*E. coli*	*Escherichia coli*
EDTA	ethylenediaminetetraacetic acid
ELISA	enzyme-linked immunosorbent assay
EMA	European Medicines Agency
Fab	fragment antigen-binding
Fc	fragment crystallizable
FDA	U.S. Food and Drug Administration
FR	framework region
Fv	variable fragment
GPCR	G protein-coupled receptor
HA	hemagglutinin (epitope tag)
HCDR3	heavy-chain complementarity-determining region 3
hFc	human IgG1 fragment crystallizable (Fc)
His	histidine
%HQ	percentage of high-quality bases
HRP	horseradish peroxidase
HTS	high-throughput sequencing
IACUC	Institutional Animal Care and Use Committee
IGHJ	immunoglobulin heavy-chain joining gene
IGHV	immunoglobulin heavy-chain variable gene
IgG	immunoglobulin G
IMGT	the international ImMunoGeneTics information system
IND	indeterminate (VH/VHH classification call)
IPTG	isopropyl β-D-1-thiogalactopyranoside
LB	Luria–Bertani
LBC	LB agar containing carbenicillin
LQ	low quality
MOPS	3-(N-morpholino)propanesulfonic acid
MPLA-SM	monophosphoryl lipid A from Salmonella minnesota R595
MQ	medium quality
NCBI	National Center for Biotechnology Information
OD405	optical density at 405 nm
OD600	optical density at 600 nm
PB	peripheral blood
PBMC	peripheral blood mononuclear cell
PBS	phosphate-buffered saline
PBST	phosphate-buffered saline containing 0.05% (*v*/*v*) TWEEN^®^ 20
PCR	polymerase chain reaction
PD-L1	programmed death-ligand 1
PEG-8000	polyethylene glycol 8000
PEI	polyethylenimine
PFU	plaque-forming unit
pIII	phage minor coat protein III
pVIII	phage major coat protein VIII
PMSF	phenylmethylsulfonyl fluoride
RBD	receptor-binding domain
RNA	ribonucleic acid
SARS-CoV-2	severe acute respiratory syndrome coronavirus 2
SB	Super Broth
SBC	Super Broth containing carbenicillin
scFv	single-chain variable fragment
sdAb	single-domain antibody
SRA	Sequence Read Archive
TES	Tris–EDTA–sucrose (buffer)
TNF-α	tumor necrosis factor-α
TU	transducing unit
VH	variable domain of the heavy chain
VHH	variable domain of the heavy chain of a heavy-chain-only antibody
VL	variable domain of the light chain
